# Maximizing Free Energy Gain

**DOI:** 10.3390/e27010091

**Published:** 2025-01-20

**Authors:** Artemy Kolchinsky, Iman Marvian, Can Gokler, Zi-Wen Liu, Peter Shor, Oles Shtanko, Kevin Thompson, David Wolpert, Seth Lloyd

**Affiliations:** 1 Department of Medicine and Life Sciences, Universitat Pompeu Fabra, 08003 Barcelona, Spain; artemyk@gmail.com; 2 Physics and Electrical Engineering, Duke University, Durham, NC 27708, USA; iman.marvian@duke.edu; 3 School of Engineering and Applied Sciences, Harvard University, Cambridge, MA 02138, USA; cgokler@gmail.com; 4 Yau Mathematical Sciences Center, Tsinghua University, Beijing 100084, China; z.w.liu.nju@gmail.com; 5 Department of Mathematics, Center for Theoretical Physics and CSAIL, MIT, Cambridge, MA 02139, USA; shor@math.mit.edu; 6 IBM Quantum Almaden, San Jose, CA 95120, USA; oles.shtanko@gmail.com; 7 Department of Physics, MIT, Cambridge, MA 02139, USA; 8 Sandia National Laboratory, Albuquerque, NM 87123, USA; kevthom@sandia.gov; 9 Santa Fe Institute, Santa Fe, NM 87501, USA; david.h.wolpert@gmail.com; 10 Center for Bio-Social Complex Systems, Arizona State University, Tempe, AZ 85287, USA; 11 Department of Mechanical Engineering, MIT, Cambridge, MA 02139, USA

**Keywords:** nonequilibrium thermodynamics, free energy, quantum mechanics

## Abstract

Maximizing the amount of work harvested from an environment is important for a wide variety of biological and technological processes, from energy-harvesting processes such as photosynthesis to energy storage systems such as fuels and batteries. Here, we consider the maximization of free energy—and by extension, the maximum extractable work—that can be gained by a classical or quantum system that undergoes driving by its environment. We consider how the free energy gain depends on the initial state of the system while also accounting for the cost of preparing the system. We provide simple necessary and sufficient conditions for increasing the gain of free energy by varying the initial state. We also derive simple formulae that relate the free energy gained using the optimal initial state rather than another suboptimal initial state. Finally, we demonstrate that the problem of finding the optimal initial state may have two distinct regimes, one easy and one difficult, depending on the temperatures used for preparation and work extraction. We illustrate our results on a simple model of an information engine.

## 1. Introduction

The last few decades have seen a revolution in non-equilibrium statistical mechanics [1,2,3,4,5,6] with the realization that many thermodynamic processes are governed by exact relations such as the Jarzynski equality [1] and the Crooks fluctuation theorem [2]. An example of such a result can be found in ref. [7], which derives expressions governing the work dissipated by a system undergoing some driven process. Specifically, a simple formula is derived that relates the minimum amount of dissipated work, versus the actual amount dissipated, as a function of the initial statistical state of the process.

In this paper, we consider a physical system that undergoes an interaction with its environment as described by a driven classical or quantum–mechanical process. By extending the result mentioned above [7], we calculate how much non-equilibrium free energy the system gains during this interaction. We also consider how the gain of free energy can be optimized by a judicious choice of the initial state. Optimizing the gain of free energy is physically meaningful, because the free energy gain sets a bound on the amount of work that the system can extract and store by interacting with a thermal environment: the maximum amount of work that can be extracted is equal to the non-equilibrium free energy minus the free energy at thermal equilibrium.

As a motivating example, consider a photosynthetic organism: before the sun rises in the morning, the organism must invest resources in order to prepare its photosynthetic machinery for harvesting free energy from the sun. When the sun sets in the evening, it stops photosynthesizing and uses the harvested free energy to survive the night, reproduce, etc. All else being equal, the organism should prepare its photosynthetic machinery in the state that maximizes the gain of free energy, since this will typically translate into higher fitness.

In the next section, we formulate our general setup and use it to calculate the free energy gain as a function of the initial state. Importantly, our calculation takes into account the preparation of the initial state as well as the extraction of free energy into a work reservoir. State preparation and work extraction may utilize external heat baths, possibly at two different temperatures.

In our first result, we derive simple necessary and sufficient conditions to guarantee that for a given interaction with the environment, free energy gain can be optimized by varying the initial state. We then derive a simple information–theoretic formula that describes the dependence of the free energy gain on the initial state. Using this formula, we relate the free energy gained when the process begins in an optimal initial state versus that gained when the process begins in a suboptimal initial state. Finally, we show that the problem of identifying the optimal initial state exhibits two distinct regimes, depending on the bath temperatures involved in state preparation versus work extraction. When work extraction happens at a lower temperature than preparation, the problem involves the maximization of a concave function, and it can be easily solved by gradient ascent. In this regime, a biological species in which each successive generation harvests more free energy is headed for the global maximum. On the other hand, when work extraction happens at a higher temperature than preparation, the objective may become nonconcave, and gradient ascent may become stuck in a suboptimal solution. At the end of this paper, we illustrate our results on an information engine.

Our results complement existing research on work extraction and free energy harvesting in classical and quantum thermodynamics [6,8,9,10,11,12,13,14,15,16]. Such research typically considers how extractable work depends on properties of the physical process—such as its speed of evolution [17,18], constrained control [19,20], or stochastic fluctuations [21,22]—given some fixed initial state. Here, we consider the complementary question of how extractable work depends on the initial state, given a fixed physical process. See also refs. [23,24,25,26,27] for related results concerning the dependence of entropy production on the initial state.

## 2. Preliminaries

We consider a physical system that harvests free energy from its environment and extracts it as work. The system may be classical or quantum, although for maximum generality, we usually employ quantum mechanical notation. For simplicity, we assume that the system is finite-dimensional, although most results can be extended to the infinite-dimensional case [24]. We will use the notation S(ρ)=−tr{ρlnρ} for the von Neumann entropy and S(ρ∥σ)=tr{ρ(lnρ−lnσ)} for the quantum relative entropy.

Our analysis will use the relationship between work and free energy for isothermal processes. Consider a process that transforms some initial state and Hamiltonian ρ,H to final state and Hamiltonian $\rho^{\prime },H^{\prime }$ while coupled to a heat bath at temperature *T*. According to the Second Law of Thermodynamics, the work that can be extracted during this transformation is bounded by the drop of *nonequilibrium free energy* [6,8,9,10,11]:

(1) $$\begin{array}{c} W\leq F_{H,T}\left(\rho \right)-F_{H^{\prime },T}\left(\rho^{\prime }\right)\;, \end{array}$$

where non-equilibrium free energy is defined as

(2) $$\begin{array}{c} F_{H,T}\left(\rho \right)=\mathrm{tr}\;\left\{\rho H\right\}-T\;S\left(\rho \right)\;. \end{array}$$

Throughout this paper, we choose energy units so that Boltzmann’s constant is $k_{B}=1$. We also use the convention that W>0 indicates work extraction while W<0 indicates work investment.

The bound (1) can be achieved in a classical system using a slow (quasistatic) driving protocol that remains close to equilibrium throughout and thus achieves thermodynamic reversibility [8,9,10]. For quantum systems, this bound is achievable by a quasistatic protocol when the two states ρ and $\rho^{\prime }$ are diagonal in their respective energy bases (as defined by *H* and $H^{\prime }$ respectively) [28]. The bound is also achievable if the quasistatic protocol operates on a large number of identical copies of the quantum system, in which case Equation (1) refers to the work per copy. In the most general case, where ρ and/or $\rho^{\prime }$ are non-diagonal and the protocol operates on a single copy of the system, the achievability of the bound remains an open question in quantum thermodynamics, possibly depending on available catalytic resources [29].

With some rearrangement, the non-equilibrium free energy can also be expressed as

(3) $$\begin{array}{c} F_{H,T}\left(\rho \right)=T\;S\left(\rho ∥\rho^{\mathrm{eq}}\right)+F_{H,T}^{\mathrm{eq}}\;, \end{array}$$

where $F_{H,T}^{\mathrm{eq}}=F_{H,T}\left(\rho^{\mathrm{eq}}\right)$ is the equilibrium free energy, which is defined using the Gibbs state $\rho^{\mathrm{eq}}=e^{-H/T}/\mathrm{tr}\;\left\{e^{-H/T}\right\}$. The first term,

(4) $$\begin{array}{c} T\;S\left(\rho ∥\rho^{\mathrm{eq}}\right)=F_{H,T}\left(\rho \right)-F_{H,T}^{\mathrm{eq}}\;, \end{array}$$

is called the *availability.* It quantifies the maximum work that can be extracted from the state ρ by bringing it to equilibrium $\rho^{\mathrm{eq}}$.

## 3. Free Energy Harvesting

**Figure 1:**
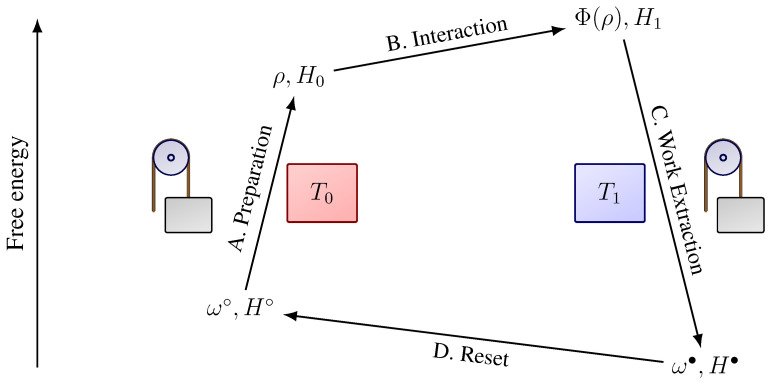
Four-stage protocol used to harvest free energy from the environment. During the Preparation stage, the system is coupled to the internal work reservoir and a heat bath at temperature $T_{0}$. During Interaction, the system harvests free energy from the external environment. During Work Extraction, the system is coupled to the internal work reservoir and a heat bath at temperature $T_{1}$. During Reset, the system is again coupled to the external environment.

Suppose that the system has access to an internal work reservoir (e.g., a battery), which is used for state preparation and work extraction. Suppose also that the system also has access to two heat baths at temperatures $T_{0}$ and $T_{1}$. The system undergoes the following four-stage procedure, which is also illustrated in Figure 1:

(A) *Preparation*: The system begins in an unprepared state $\omega^{\circ }$ and Hamiltonian $H^{\circ }$. It is then driven to the prepared state ρ and Hamiltonian $H_{0}$ while coupled to the internal work reservoir and heat bath at temperature $T_{0}$. Given Equation (1), the work extracted during this transformation is bounded by
  (5) $$\begin{array}{c} W_{A}\leq F_{H^{\circ },T_{0}}\left(\omega^{\circ }\right)-F_{H_{0},T_{0}}\left(\rho \right)\;. \end{array}$$
(B) *Interaction/Free energy harvesting*: The system is disconnected from the work reservoir. It then undergoes a fixed interaction with the environment, which may contain any number of thermodynamic reservoirs, free energy sources, and external work reservoirs (e.g., the sun). At the end of this stage, the system has Hamiltonian $H_{1}$ and state Φ(ρ). Here, Φ is the quantum channel (completely positive and trace-preserving map) that describes the system’s evolution due to the interaction with the environment.
(C) *Work Extraction*: The system is coupled to the work reservoir and the heat bath at temperature $T_{1}$. It is then driven from state Φ(ρ) and Hamiltonian $H_{1}$ to final state $\omega^{•}$ and Hamiltonian $H^{•}$. According to Equation (1), the maximum work that can be extracted during this transformation is
  (6) $$\begin{array}{c} W_{C}\leq F_{H_{1},T_{1}}\left(\Phi \left(\rho \right)\right)-F_{H^{•},T_{1}}\left(\omega^{•}\right)\;. \end{array}$$
(D) *Reset*: The system is disconnected from the internal work reservoir and then undergoes another interaction with the environment. As a result of this interaction—which, in some cases, may be a simple relaxation — the system ends in state $\omega^{\circ }$ and Hamiltonian $H^{\circ }$. This completes the cycle, thereby preparing the system for Stage A. In the special case where $\omega^{•}=\omega^{\circ }$ and $H^{•}=H^{\circ }$, the Reset stage is not necessary.

As a concrete—though still highly idealized—example of our setup, one might imagine a simple photosynthetic system, such as Bacteriorhodopsin in archaea [20,30]. During Preparation, the organism spends free energy (by hydrolyzing ATP) to synthesize the Bacteriorhodopsin protein from free-floating amino acids. During Interaction, the protein uses solar energy to pump protons across the cellular membrane, thereby increasing the membrane potential. During Work Extraction, the membrane potential is used by ATPase to synthesize ATP. Reset may occur by consumption of any additional ATP and degradation of the Bacteriorhodopsin protein back into amino acids. We note that in this system, Preparation and Work Extraction steps may occur at different temperatures.

We now calculate a bound on the work that can be extracted using this four-stage process. Since the system only interacts with the work reservoir during Stages A and C, the total amount of extracted work is

(7) $$\begin{array}{c} W=W_{C}+W_{A}. \end{array}$$

Equations (5) and (6) then imply the upper bound W≤G(ρ), where we define

(8) $$\begin{array}{c} G\left(\rho \right)=\left[F_{H_{1},T_{1}}\left(\Phi \left(\rho \right)\right)-F_{H_{0},T_{0}}\left(\rho \right)\right]-\left[F_{H^{•},T_{1}}\left(\omega^{•}\right)-F_{H^{\circ },T_{0}}\left(\omega^{\circ }\right)\right]\;. \end{array}$$

Observe that G(ρ) is a function of the initial state and that it consists of two terms. The first term is the gain of non-equilibrium free energy during the Interaction with the environment (Stage B). The second term is the loss of non-equilibrium free energy during the Reset (Stage D).

We may also rewrite G(ρ) in the following form:

(9) $$\begin{array}{cc} G(\rho ) & =T_{1}\;S\left(\Phi \left(\rho \right)∥\pi_{1}\right)-T_{0}\;S\left(\rho ∥\pi_{0}\right)+G_{\mathrm{base}}\;, \end{array}$$

where $\pi_{0}=e^{-H_{0}/T_{0}}/\mathrm{tr}\left\{e^{-H_{0}/T_{0}}\right\}$ and $\pi_{1}=e^{-H_{1}/T_{1}}/\mathrm{tr}\left\{e^{-H_{1}/T_{1}}\right\}$ refer to Gibbs states corresponding to $\left(H_{0},T_{0}\right)$ and $\left(H_{1},T_{1}\right)$ respectively.

This expression follows by combining Equations (3) and (8) and rearranging, while also defining the “baseline” term

(10) $$\begin{array}{cc} G_{\mathrm{base}} & =\left[F_{H^{\circ },T_{0}}\left(\omega^{\circ }\right)-F_{H_{0},T_{0}}^{\mathrm{eq}}\right]-\left[F_{H^{•},T_{1}}\left(\omega^{•}\right)-F_{H_{1},T_{1}}^{\mathrm{eq}}\right] \end{array}$$

Observe that Equation (9) expresses G(ρ) as the gain of availability, Equation (4), during the transition from ρ to Φ(ρ), plus a constant offset ($G_{\mathrm{base}}$).

In the following, we will generally be interested in the dependence of G(ρ) on the initial state ρ. In Equation (8), this dependence is captured by first term, the gain of non-equilibrium free energy, since the second term does not depend on ρ (nor on the channel Φ). In Equation (9), this dependence is captured entirely by the gain of availability, since $G_{\mathrm{base}}$ again does not depend on ρ (nor on the channel Φ).

For convenience, we will often refer to G(ρ) simply as the “free energy gain”.

## 4. Increasing Free Energy Gain

We now consider the problem of maximizing the free energy gain G(ρ) with respect to the initial state ρ. Before proceeding, we note that maximizing free energy gain is not always the same as maximizing extracted work *W*. The two optimization problems are equivalent when the the Preparation and Work Extraction stages are thermodynamically reversible, so that the bounds (5) and (6) are saturated. The optimization of free energy gain G(ρ) is relevant when Preparation and Work Extraction stages are thermodynamically optimal. Moreover, because G(ρ) always sets an upper bound on extractable work, maximizing G(ρ) is also relevant when the precise details of Preparation and Work Extraction are unknown, varying, or simply undefined.

To study the problem of optimizing G(ρ), we first consider a few special cases. First, suppose that Φ is a (generalized) Gibbs-preserving map that transforms the initial Gibbs state to the final Gibbs state $\pi_{1}$, $\Phi \left(\pi_{0}\right)=\pi_{1}$. This situation applies if the driving by the environment is sufficiently slow so that the system remains in equilibrium throughout (quasistatic driving), or if the system is allowed to equilibrate at the end of its interaction with the environment. Then, by monotonicity of relative entropy [31],

(11) $$\begin{array}{c} S\left(\Phi \left(\rho \right)∥\pi_{1}\right)=S\left(\Phi \left(\rho \right)∥\Phi \left(\pi_{0}\right)\right)\leq S\left(\rho ∥\pi_{0}\right)\;. \end{array}$$

Combining with Equation (9) implies that $G\left(\rho \right)\leq G_{\mathrm{base}}$ for all ρ whenever $T_{1}\leq T_{0}$. That is, if Φ maps $\pi_{0}$ to $\pi_{1}$ and the temperature of Work Extraction is less than or equal to the temperature of Preparation, it is impossible to extract more work than $G_{\mathrm{base}}$ regardless of the initial state. Moreover, since $G\left(\pi_{0}\right)=G_{\mathrm{base}}$, one cannot do better than the naive strategy of setting the initial state to $\pi_{0}$, i.e., letting the system relax fully to equilibrium for Hamiltonian $H_{0}$ and temperature $T_{0}$.

On the other hand, suppose that Φ is not Gibbs preserving, so $\Phi \left(\pi_{0}\right)\neq \pi_{1}$. Suppose we still choose the initial state as $\pi_{0}$. Equation (9) then gives

(12) $$\begin{array}{c} G\left(\pi_{0}\right)=T_{1}\;S\left(\Phi \left(\pi_{0}\right)∥\pi_{1}\right)+G_{\mathrm{base}}>G_{\mathrm{base}}\;, \end{array}$$

where the last inequality follows from the positivity of the relative entropy between different states. Thus, if Φ is not Gibbs preserving, there is always at least one initial state for which G is strictly greater than $G_{\mathrm{base}}$. Moreover, generally, G can be increased even further by optimizing the choice of the initial state.

These conditions are formalized by the following statement.

**Theorem 1.** 

*(1a) If $\Phi \left(\pi_{0}\right)=\pi_{1}$, there exists some ρ with $G\left(\rho \right)>G_{\mathrm{base}}$ only if $T_{1}>T_{0}$.*
*(1b) If $\Phi \left(\pi_{0}\right)\neq \pi_{1}$, there always exists some ρ with $G\left(\rho \right)>G_{\mathrm{base}}$.*

As a special case, consider the situation where the quantum channel is the identity (Φ(ρ)=ρ for all inputs) and the Hamiltonians $H_{0},H_{1}$ are equal. In that case, $\Phi \left(\pi_{0}\right)=\pi_{1}$ only if $T_{0}=T_{1}$. Then, Theorem 1 implies that free energy can be gained beyond $G_{\mathrm{base}}$ if and only if $T_{0}\neq T_{1}$. In this special case, the interaction with the environment provides no free energy, so free energy can only be gained using a temperature difference between the baths, as in a heat engine.

## 5. Dependence on the Initial State

We now consider how the free energy gain depends on the choice of the initial state. Before proceeding, we provide a useful expression for the (one-sided) directional derivative of G. Recall that the directional derivative at state σ toward state ρ is defined as

(13) $$D_{\rho -\sigma }G\left(\sigma \right)=\lim_{\lambda \to 0^{+}}\frac{G[\sigma +\lambda (\rho -\sigma )]-G(\sigma )}{\lambda }$$

In Appendix A, we show that this directional derivative can be expressed as

(14) $$\begin{array}{c} D_{\rho -\sigma }G\left(\sigma \right)=G\left(\rho \right)-G\left(\sigma \right)+T_{0}\;S\left(\rho ∥\sigma \right)-T_{1}\;S\left[\Phi \left(\rho \right)∥\Phi \left(\sigma \right)\right]\;. \end{array}$$

This expression is particularly useful when considering σ for which the directional derivatives vanishes. This is shown in the following result, which is proved in Appendix B.

**Theorem 2.**  *Let σ be a (local or global) minimum, maximum, or saddle point of G. Then, for any ρ with S(ρ∥σ)<∞,*

(15) $$\begin{array}{c} G\left(\sigma \right)-G\left(\rho \right)=T_{0}\;S\left(\rho ∥\sigma \right)-T_{1}\;S\left[\Phi \left(\rho \right)∥\Phi \left(\sigma \right)\right]. \end{array}$$

Theorem 2 means that the increase in free energy gain when using initial state σ versus ρ has a universal information-theoretic expression. While the left-hand side of Equation (15) contains thermodynamic terms, the right-hand side of this equality consists purely of information-theoretic quantities, that is relative entropies, scaled by the temperatures. The change in the relative entropies can be understood as the loss of distinguishability between ρ and σ during the process, and it does not explicitly depend on the energy functions. Indeed, Theorem 2 provides a simple example of a relationship between information-theoretic and physical quantities. Such relationships have been found to be very useful in the resource theory of thermodynamics [12,14,32,33].

## 6. Optimal Initial State

We now consider the problem of optimizing the initial state so as to maximize free energy gain. Consider the initial state σ that is a local or global maximizer of G. Then, according to Theorem 2, for any other initial state ρ with S(ρ∥σ)<∞,

(16) $$\begin{array}{c} G\left(\sigma \right)-G\left(\rho \right)=T_{0}\;S\left(\rho ∥\sigma \right)-T_{1}\;S\left[\Phi \left(\rho \right)∥\Phi \left(\sigma \right)\right] \end{array}$$

Equation (16) provides a simple formula for computing the free energy gain that is lost when the system is prepared in the “wrong” initial state: it is given by comparing the relative entropy between the “wrong” and “right” (i.e., optimal) states at the beginning of the process with the the same relative entropy at the end of the process, multiplied by the temperatures $T_{0}$ and $T_{1}$.

Next, we consider the difficulty of finding the optimal state σ. As we now show, G is a concave function of the initial state if the temperature of Preparation is no cooler than the temperature of Work Extraction. The proof is found in Appendix B.

**Theorem 3.**  *G(ρ) is a concave function of ρ if $T_{0}\geq T_{1}$.*

Any local maximizer of a concave function must also be a global maximizer. Thus, Theorem 3 implies that as long as $T_{0}\geq T_{1}$, the global optimization of free energy harvesting can be accomplished by a simple procedure, e.g., gradient ascent in the space of density matrices [25]. Consider an adaptive system that undergoes the same free energy harvesting process many times. Each time the system goes through the free energy harvesting cycle, it has the opportunity to vary its initial state ρ to try to increase the free energy gain. The concavity of free energy maximization implies that if the adaptive process is able to alter the initial state to improve the amount of free energy harvested in each round, then the adaptive system is headed for the global optimum, and will not become stuck in a local optimum.

A population of photosynthetic bacteria, for example, exhibits genetic variation in the individuals’ molecular mechanisms for performing the Preparation, Interaction, and Work Extraction stages, all of which impact the viability of the individual organisms and their ability to reproduce. In general, we expect that the more efficient an individual bacterium is at harvesting free energy, the more viable it will be, resulting in its offspring forming a larger fraction of the population in subsequent generations. Focusing only on the free energy harvesting stage, we see that genetic variations which increase the amount of free energy harvested—e.g., a small change in the structure of a photo-harvesting chromophore which provides greater overlap with the absorptive spectrum of the chromophore and ambient light conditions—will guide the population as a whole to adapt its composition to become more efficient at energy harvesting.

The concavity of free energy gain as a function of the initial state of the bacteria implies that if the free energy harvesting is suboptimal, then there is always a nearby initial state that improves the free energy gain. The only way for the adaptive process to become stuck in a local optimum is if the genetic variation in the population is unable to explore fully the space of initial probabilistic states.

If Work Extraction occurs at a warmer temperature than Preparation, however, in general, there is no guarantee that G is concave. In this case, finding the global optimum may be a much harder problem, and gradient ascent may become trapped in suboptimal local maxima.

Finally, we note that Equation (16) only holds for those ρ that obey S(ρ∥σ)<∞. This condition is equivalent to the requirement that the support of ρ falls within the support of σ. For this reason, the applicability of Equation (16) is most general in those cases where the optimizer σ has full support. In our final result, we provide some simple sufficient conditions for σ to have full support. The proof is found in Appendix B.

**Theorem 4.**  *Any (local or global) maximizer σ of G has full support if $T_{0}>0$ and Φ(ρ) has full support for all ρ, or if $T_{0}>T_{1}$.*

## 7. Example

We illustrate our results using a simple example of a two-level system. The system can be considered as a model of an “information engine” which gains free energy by interacting with a heat bath and a low-entropy environment [34,35].

For simplicity, we begin by focusing on the classical case, where all Hamiltonians, states, and channels are diagonal in the same basis. We use classical notation *p* (instead of ρ) to indicate the actual initial probability distribution of the system, *q* (instead of σ) to indicate the optimal initial distribution, and *P* (instead of Φ) to indicate the classical transition matrix (conditional probability of outputs given inputs). We also use the notation $D\left(p∥q\right)=\sum_{x}p\left(x\right)\ln\left[p(x)/q(x)\right]$ to indicate the classical relative entropy (also known as Kullback–Leibler divergence) and $H\left(p\right)=-\sum_{x}p\left(x\right)\ln p(x)$ for the Shannon entropy.

The engine is modeled as an overdamped two-level system X∈{0,1} with energy gap ϵ≥0, with energy function H(0)=0,H(1)=ϵ. The engine is coupled to the *environment*, another two-level system Y∈{0,1} with a uniform energy function, $H_{\mathrm{env}}\left(0\right)=H_{\mathrm{env}}\left(1\right)=0$. The engine and environment are weakly coupled, so their joint energy function can be decomposed as $H_{\mathrm{tot}}\left(x,y\right)\approx H\left(x\right)+H_{\mathrm{env}}\left(y\right)$. Also, initially at time t=0, the engine and the environment are statistically independent: $p_{\mathrm{tot}}\left(x,y\right)=p\left(x\right)p_{\mathrm{env}}\left(y\right)$. Then, over the time interval t∈[0,τ], the two systems relax freely while connected to a heat bath at temperature T=1. The environment and the engine have coupled transitions: the 0→1 transition in the engine occurs only when the environment simultaneously undergoes a 0→1 transition, and this is vice versa for the 1→0 transition. No transitions occur in/out of microstates where the engine and environment occupy different levels, (x,y)∈{(0,1),(1,0)}.

Suppose the engine is used to extract work using the four-stage protocol shown in Figure 1. We assume that Stage A (Preparation) starts and ends with the same Hamiltonian ($H^{\circ }=H_{0}=H$), and that the unprepared state is the Gibbs state $\pi_{0}$ at some temperature $T_{0}$ ($\omega^{\circ }=\pi_{0}$). During Stage B (Interaction), the engine evolves according to a transition matrix *P*, which is defined below. Finally, we assume Stage C (Work Extraction) starts and ends with the same Hamiltonian ($H_{1}=H^{•}=H$) and that the final state is the Gibbs state $\pi_{1}$ at some temperature $T_{1}$.

The net amount of extracted work is bounded W≤G(p) by the gain of availability:

(17) $$\begin{array}{c} G\left(p\right)=T_{1}\;D\left(Pp∥\pi_{1}\right)-T_{0}\;D\left(p∥\pi_{0}\right) \end{array}$$

This result follows from Equation (9) and the fact that $G_{\mathrm{base}}=0$ (given our assumptions). At the same time, our results imply that G can be expressed as

(18) $$\begin{array}{c} G\left(p\right)=G\left(q\right)-\left[T_{0}\;D\left(p∥q\right)-T_{1}\;D\left(Pp∥Pq\right)\right]\;, \end{array}$$

where $q\in {arg max}_{p}G\left(p\right)$ is a maximizer of G, as given in Equation (16), which is valid whenever *q* has full support. A simple sufficient condition for *q* to have full support is for environment distribution $p_{\mathrm{env}}$ to have full support; this follows from Theorem 4 and because then Pp has full support for all *p* (see Equation (21) below). The function G and the optimizer *q* will depend on the parameters of the problem: the energy gap ϵ, the initial environment distribution $p_{\mathrm{env}}$, and the temperature of Preparation ($T_{0}$), Interaction (*T*), and Work Extraction ($T_{1}$).

To construct *P*, we assume that the engine and environment undergo continuous-time Markovian dynamics, which are represented by the rate matrix

$$\begin{array}{c} R=\left[\begin{array}{cccc} -1 & 0 & 0 & e^{\epsilon }\\ 0 & 0 & 0 & 0\\ 0 & 0 & 0 & 0\\ 1 & 0 & 0 & -e^{\epsilon } \end{array}\right]\;, \end{array}$$

where $R_{ij}$ is the transition rate from state *j* to state *i* with the four states referring to (x,y)={(0,0), (0,1), (1,0),(1,1)}. *R* obeys local detailed balance for the energy function $H_{\mathrm{tot}}$ and interaction temperature T=1. We consider the limit of a long relaxation, corresponding to the following joint transition matrix:

(19) $$\begin{array}{c} P_{\mathrm{tot}}=\lim_{\tau \to \infty }e^{\tau R}=\left[\begin{array}{cccc} \frac{1}{1+e^{-\epsilon }} & 0 & 0 & \frac{1}{1+e^{-\epsilon }}\\ 0 & 1 & 0 & 0\\ 0 & 0 & 1 & 0\\ \frac{e^{-\epsilon }}{1+e^{-\epsilon }} & 0 & 0 & \frac{e^{-\epsilon }}{1+e^{-\epsilon }} \end{array}\right]\;. \end{array}$$

The transition matrix of the engine subsystem *X* is computed by marginalizing

(20) $$\begin{array}{c} P\left(x^{\prime }|x\right)=\sum_{y,y^{\prime }}P_{\mathrm{tot}}\left(x^{\prime },y^{\prime }|x,y\right)p_{\mathrm{tot}}\left(y|x\right)=\sum_{y,y^{\prime }}P_{\mathrm{tot}}\left(x^{\prime },y^{\prime }|x,y\right)p_{\mathrm{env}}\left(y\right)\;, \end{array}$$

and it can written explicitly in matrix notation as

(21) $$\begin{array}{c} P=\frac{1}{1+e^{-\epsilon }}\left[\begin{array}{cc} 1+e^{-\epsilon }-p_{\mathrm{env}}\left(0\right)e^{-\epsilon } & 1-p_{\mathrm{env}}\left(0\right)\\ p_{\mathrm{env}}\left(0\right)e^{-\epsilon } & p_{\mathrm{env}}\left(0\right)+e^{-\epsilon } \end{array}\right] \end{array}$$

**Figure 2:**
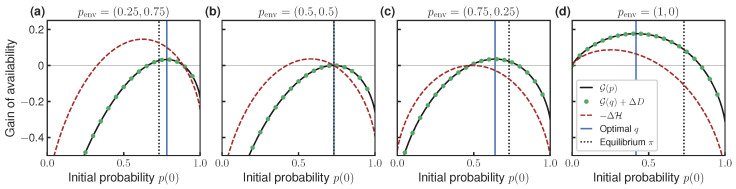
Availability gain G(p) as a function of the engine initial distribution (p(0),1−p(0)). (**a**–**d**) correspond to four different environment initial distributions $p_{\mathrm{env}}$. Black lines show G(p) computed using Equation (17); green dots indicate predictions made using our information-theoretic expression (18) (using shorthand G(q)+ΔD in legend). Optimal initial distribution *q* and equilibrium initial distribution π are indicated using vertical lines. Dashed curve indicates reduction in the engine’s Shannon entropy as a function of initial distribution, −ΔH from Equation (22). Vertical axes have the same scale. Other parameters: $T_{0}=T_{1}=T=1$, ϵ=1.

We now illustrate our results with some numerical experiments. In Figure 2, we show the gain of availability G(p) as a function of the engine’s initial distribution *p*, which is computed using Equation (17). Since the engine only has two microstates, *p* is fixed by probability p(0) of microstate X=0, as shown on the horizontal axis. The different subplots correspond to the different initial distribution of the environment $p_{\mathrm{env}}$. The other parameters are set to ϵ=1 and $T_{0}=T_{1}=T=1$. We show the location of the optimal initial distribution *q* found by numerical optimization, and the value of G(p) is computed using using our information-theoretic expression (17). We also show the location of the initial equilibrium distribution $\pi_{0}=\left(\frac{1}{1+e^{-\epsilon }},\frac{e^{-\epsilon }}{1+e^{-\epsilon }}\right)$.

We comment on some interesting aspects of the results in Figure 2. First, G is a concave function of the initial distribution, which is in accordance with Theorem 3. We also verify our main result, showing that the thermodynamic (17) and information-theoretic (18) expressions for G are equivalent. Note that maximum availability gain is non-monotonic in $p_{\mathrm{env}}\left(0\right)$. In Figure 2b, the environment starts from the maximum entropy state $p_{\mathrm{env}}=\left(0.5,0.5\right)$; in this case, the optimal initial distribution is the equilibrium one ($q=\pi_{0}$), and it is not possible to harvest strictly positive availability (G(q)=0). On the other hand, strictly positive availability can be harvested when the environment is biased to microstate Y=0, see Figure 2a, or Y=1, see Figure 2c,d, but the optimal strategy differs in these two cases. When the environment is biased to Y=1 ($p_{\mathrm{env}}\left(0\right)<0.5$), the optimal initial distribution is biased to X=0 relative to equilibrium (q(0)>π(0)). Conversely, when the environment is biased to Y=0 ($p_{\mathrm{env}}\left(0\right)<0.5$), the optimal initial distribution is biased toward X=1 relative to equilibrium (q(0)<π(0)). This reflects the balance between two effects. On one hand, there is an advantage to biasing the engine’s initial distribution toward X=0, because the transition 0→1 harvests ϵ energy from the heat bath. On the other hand, availability can also be harvested by decreasing the Shannon entropy of the engine, i.e., by increasing

(22) $$\begin{array}{c} -\Delta H:=T_{0}H\left(p\right)-T_{1}H\left(Pp\right)\;. \end{array}$$

This quantity is shown as the dashed red line in Figure 2. It can be seen that this second effect shifts the optimal distribution toward X=1 relative to equilibrium, and it becomes stronger when the environment is more concentrated on state Y=1.

Figure 2d shows that *q* has full support even though $p_{\mathrm{env}}=\left(1,0\right)$ does not have full support (so Pp does not have full support for all *p*; see Equation (21)). This demonstrates that Theorem 4 is only a sufficient, but not necessary, condition for the optimizer *q* to have full support.

**Figure 3:**
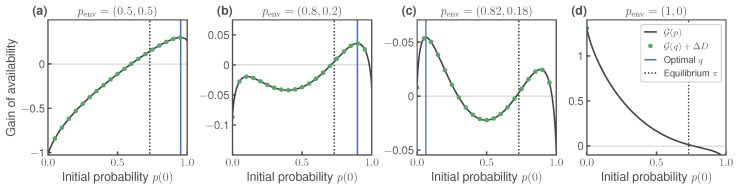
Same as in Figure 2, but where the temperature of Work Extraction is higher than of Preparation, $T_{1}=3>T_{0}=1$. Black lines show G(p) computed using Equation (17); green dots indicate predictions made using information-theoretic expression (18). (**a**–**d**) correspond to different initial states of the environment. Observe that in some cases, the function G is non-concave and may have multiple local maxima. In (**d**), the optimal distribution *q* does not have full support, so the equivalence between Equations (17) and (18) does not hold.

In Figure 3, we consider the same system but now setting the temperature of Work Extraction higher than that of Preparation and Interaction, $T_{0}=T=1,T_{1}=3$. As above, different subplots correspond to different initial distributions of the environment. To emphasize interesting features, we make several changes with respect to Figure 2: we explore different initial environment distributions, the scale of the y-axes are different, and for simplicity, we do not show the change of Shannon entropy −ΔH.

When $T_{1}>T_{0}$, Theorem 3 no longer applies and the function G(p) may become non-concave, as seen in Figure 3b–d. Moreover, Figure 3b,c show that G may even have multiple local maxima. Note that in these two plots, the shape of G changes and the identity of the higher local maxima switches, even though $p_{\mathrm{env}}$ undergoes a very small change. In Figure 3a–c, we verify that the thermodynamic (17) and information-theoretic (18) expressions for G are equivalent. For systems with several local maxima/minima, we verified that this equivalence holds regardless of which critical point is chosen as *q*. Note that $T_{1}>T_{0}$ is necessary but not sufficient for non-concavity, since G remains concave in Figure 3a. Also, Figure 3d, $p_{\mathrm{env}}$ does not have full support and Theorem 4 no longer holds. In this case, the optimal initial distribution q=(0,1) does not have full support, so our information-theoretic expression no longer applies.

In our last numerical experiment, we consider this system in the quantum regime. We define a quantum channel Φ that dephases any input state ρ in the reference basis {|0〉,|1〉}; then, we apply the transition matrix *P* in this basis:

(23) $$\begin{array}{c} \Phi \left(\rho \right)=\sum_{x\in \left\{0,1\right\},x^{\prime }\in \left\{0,1\right\}}P_{x^{\prime }x}|x^{\prime }\rangle \langle x^{\prime }|\langle x|\rho |x\rangle . \end{array}$$

The Hamiltonians used for Preparation and Work Extraction are allowed to be coherent (non-diagonal) with respect to the reference basis. Specifically, we consider the same Hamiltonian with energy gap ϵ but rotated by angle θ with respect to the reference basis:

(24) $$\begin{array}{c} H^{\circ }=H_{0}=H_{1}=H^{•}=\epsilon U_{\theta }\left|1\rangle \langle 1\right|U_{\theta }^{†}\;\;U_{\theta }=\left[\begin{array}{cc} \cos\theta & -\sin\theta \\ \sin\theta & \cos\theta \end{array}\right] \end{array}$$

The net amount of extracted work is bounded by the gain of availability:

(25) $$\begin{array}{c} G\left(\rho \right)=T_{1}\;S\left(\Phi \left(\rho \right)∥\pi_{1}\right)-T_{0}\;S\left(p∥\pi_{0}\right) \end{array}$$

where $\pi_{0}$ and $\pi_{1}$ indicate Gibbs states for $\left(H_{0},T_{0}\right)$ and $\left(H_{1},T_{1}\right)$, respectively. As above, our results imply that G can also be expressed as

(26) $$\begin{array}{c} G\left(\rho \right)=G\left(\sigma \right)-\left[T_{0}\;S\left(\rho ∥\sigma \right)-T_{1}\;S\left(\Phi \left(\rho \right)∥\Phi \left(\sigma \right)\right)\right]\;, \end{array}$$

where $\sigma \in {arg max}_{\rho }G\left(\rho \right)$ is a maximizer of G. Equations (25) and (26) are simply the quantum versions of Equations (17) and (18).

**Figure 4:**
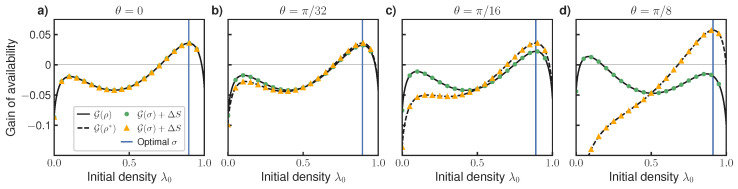
Gain of availability G(ρ) in a quantum system for different amounts of coherence (parameterized by θ). Solid black line shows G for states diagonal in the reference basis, dashed black line shows G for states diagonal in the basis of the optimizer σ, both calculated using Equation (25). Markers indicate predicted values of G from information-theoretic expression (26). (**a**) For θ=0 (no coherence), we recover the classical result shown in Figure 3b. (**b**–**d**) Advantage of selecting initial state in the optimal basis increases with increased coherence. Vertical axes have the same scale. See text for details.

Figure 4 shows the results for four values of θ, which varies the amount of coherence. The other parameters are chosen as in Figure 3b ($T_{0}=T=1,T_{1}=3,\epsilon =1,p_{\mathrm{env}}=\left(0.8,0.2\right)$). For each θ, we use Equation (25) to calculate the value of G for two sets of states ρ, which are always indexed by the (lower energy) eigenvalue $\lambda_{0}$. First, solid lines indicate G for states ρ diagonal in the reference basis, $\rho =\lambda_{0}\left|0\rangle \langle 0\right|+\left(1-\lambda_{0}\right)\left|1\rangle \langle 1\right|$. Second, dashed lines indicate G for states $\rho^{*}$ diagonal in the same basis as the optimal initial state σ. Vertical lines indicate the location of σ in this optimal basis. We also plot the values of G calculated using our information-theoretic expression Equation (26), verifying that it matches those calculated using Equation (25) for both sets of states.

There is no coherence in Figure 4a, so we effectively recover the classical case shown in Figure 3b. For higher θ>0, Figure 4b–d demonstrate that availability gain can be increased by preparing the initial state in the correct basis. Moreover, we verified that the optimal state σ is not diagonal in either the reference basis nor the basis of the rotated Hamiltonian $H_{0}=H_{1}$. The optimal basis arises from the balance of two effects: the cost of preparation (which favors σ being in the same basis as *H*) versus the free energy dissipated when σ is dephased by Φ (which favors σ being in the reference basis). Under the optimal strategy, the engine gains availability not only by increasing energy or decreasing entropy but also by harvesting coherence with respect to the basis of $H_{0}=H_{1}$.

## 8. Discussion

In this paper, we investigated how the gain of free energy depends on the initial state while considering a broad class of classical and quantum process. We first showed that the initial state can be used to optimize the gain of free energy if and only if a process fails to map equilibrium distributions to equilibrium distributions. We also derived information-theoretic formulae for the gain of free energy as a function of the initial state, and we then used these to quantify the difference in free energy gain between the optimal initial state and some suboptimal initial state. This difference was shown to be equal to the drop of the relative entropy between the initial and final states, which were scaled by temperatures.

For macroscopic systems, the deficit in free energy harvested by a suboptimal initial state may itself be a macroscopic quantity. Moreover, for a living system that requires free energy to survive and reproduce, there is considerable evolutionary pressure to increase the amount of free energy harvested. The difficulty of maximizing this objective depends on whether it is concave or not. We derived conditions for the free energy gain to be a concave function of the initial state. When these conditions hold, the objective can be maximized using a simple strategy like gradient ascent; for example, a species where each generation does slightly better at harvesting free energy will eventually approach the global maximum. In cases where the conditions do not hold, the free energy gain may become nonconcave. In such cases, the maximization of the objective becomes qualitatively more difficult, and simple strategies like gradient ascent may become trapped in local optima.

As mentioned in Section 4 above, the optimization of free energy gain is not necessarily the same as optimization of extracted work *W*, although the two problems become equivalent when the Preparation and Work Extraction stages are thermodynamically reversible. When the Preparation and Work Extraction stages are not reversible, the state that maximizes extracted work—for example, as might be found varying some control parameters of the Preparation stage (assuming all else held fixed)—is not necessarily the same as the state that maximizes free energy gain. An interesting direction for future research would consider the optimization of extracted work, assuming some realistic constraints on Preparation and/or Work Extraction protocols, e.g., constraints on preparable initial states or finite-time constraints.

## Data Availability

No new data were created or analyzed in this study. Data sharing is not applicable to this article.

## Appendix A. Derivation of Directional Derivative, Equation (14)

Using Equations (9) and (13), we may write the directional derivative of G as

(A1) $$\begin{array}{c} D_{\rho -\sigma }G\left(\sigma \right)=T_{1}\;D_{\Phi (\rho )-\Phi (\sigma )}S\left(\Phi \left(\sigma \right)∥\pi_{1}\right)-T_{0}\;D_{\rho -\sigma }S\left(\sigma ∥\pi_{0}\right)\;, \end{array}$$

where the directional derivatives of the relative entropy terms are with respect to the first argument. The directional derivative of the relative entropy $S(\sigma ∥\pi_{0})$ is [36] (Lemma 1)

$$\begin{array}{c} D_{\rho -\sigma }S\left(\sigma ∥\pi_{0}\right)=\mathrm{tr}\left\{\left(\rho -\sigma \right)\left(\ln\sigma -\ln\pi_{0}\right)\right\}\;. \end{array}$$

Rearranging the right-hand side gives

$$\begin{array}{cc} D_{\rho -\sigma }S\left(\sigma ∥\pi_{0}\right) & =S\left(\rho ∥\pi_{0}\right)-S\left(\rho ∥\sigma \right)-S\left(\sigma ∥\pi_{0}\right)\;. \end{array}$$

Similarly, the directional derivative of the relative entropy $S(\;\cdot \;∥\pi_{1})$ obeys

$$\begin{array}{cc} D_{\rho -\sigma }S\left(\Phi \left(\sigma \right)∥\pi_{1}\right) & =S\left[\Phi \left(\rho \right)∥\pi_{1}\right]-S\left[\Phi \left(\rho \right)∥\Phi \left(\sigma \right)\right]-S\left[\Phi \left(\sigma \right)∥\pi_{1}\right]\;. \end{array}$$

Plugging these expressions into Equation (A1) gives

$$\begin{array}{cc} D_{\rho -\sigma }G\left(\sigma \right) & =T_{1}\left[S\left[\Phi \left(\sigma \right)∥\pi_{1}\right]-S\left[\Phi \left(\rho \right)∥\Phi \left(\sigma \right)\right]-S\left[\Phi \left(\rho \right)∥\pi_{1}\right]\right]\\ \; & \;-T_{0}\left[S\left(\rho ∥\pi_{0}\right)-S\left(\rho ∥\sigma \right)-S\left(\sigma ∥\pi_{0}\right)\right]. \end{array}$$

Finally, we plug in the definitions of G(σ) and G(ρ) from Equation (9) and rearrange to give Equation (14).

## Appendix B. Proofs

**Proof of Theorem 2.** If S(ρ∥σ)<∞, then the support of ρ falls within the support of σ, which in turn implies that σ−αρ is positive-definite for some α>0 [37] (p. 15). Therefore, it is possible to move along the line σ+λ(ρ−σ) both toward ρ (λ>0) and away from ρ (λ<0). If σ is a minimum (or maximum), the directional derivative $D_{\rho -\sigma }G\left(\sigma \right)$ must vanish, since otherwise, G could be decreased (or increased) by a small perturbation toward or away from ρ. On the other hand, if σ is a saddle point, the directional derivative vanishes by definition. The result follows by plugging $D_{\rho -\sigma }G\left(\sigma \right)=0$ into Equation (14) and rearranging. □

**Proof of Theorem 3.** For any pair of states ρ and σ, let $\sigma_{\lambda }:=\left(1-\lambda \right)\sigma +\lambda \rho$ refer to the convex mixture with weight λ∈[0,1]. Next, we define the following quantity:

$$\begin{array}{cc} \chi & =\left(1-\lambda \right)G\left(\sigma \right)+\lambda G\left(\rho \right)-G\left(\sigma_{\lambda }\right)\;, \end{array}$$

which measures the degree of convexity G over the states $\left\{\sigma_{\lambda }:\lambda \in \left[0,1\right]\right\}$. It is positive when G is convex, negative when concave, and zero when linear. Using Equation (9), we may express χ as

(A2) $$\begin{array}{c} \begin{array}{cc} \chi & =T_{1}\left[\left(1-\lambda \right)S\left(\Phi \left(\sigma \right)∥\pi_{1}\right)+\lambda S\left(\Phi \left(\rho \right)∥\pi_{1}\right)-S\left(\Phi \left(\sigma_{\lambda }\right)∥\pi_{1}\right)\right]\\ \; & \;-T_{0}\left[\left(1-\lambda \right)S\left(\sigma ∥\pi_{0}\right)+\lambda S\left(\rho ∥\pi_{0}\right)-S\left(\sigma_{\lambda }∥\pi_{0}\right)\right] \end{array} \end{array}$$

The bracketed term in second line can be rearranged as

$$\begin{array}{c} \left(1-\lambda \right)S\left(\sigma ∥\pi_{0}\right)+\lambda S\left(\rho ∥\pi_{0}\right)-S\left(\sigma_{\lambda }∥\pi_{0}\right)=\left(1-\lambda \right)S\left(\sigma ∥\sigma_{\lambda }\right)+\lambda S\left(\rho ∥\sigma_{\lambda }\right) \end{array}$$

In a similar way, the bracketed term in the first line can be rearranged as

$$\begin{array}{c} \left(1-\lambda \right)S\left(\Phi \left(\sigma \right)∥\Phi \left(\sigma_{\lambda }\right)\right)+\lambda S\left(\Phi \left(\rho \right)∥\Phi \left(\sigma_{\lambda }\right)\right) \end{array}$$

Plugging back into Equation (A2) gives

$$\begin{array}{cc} \chi & =\left(1-\lambda \right)\left[T_{1}\;S\left(\Phi \left(\sigma \right)∥\Phi \left(\sigma_{\lambda }\right)\right)-T_{0}S\left(\sigma ∥\sigma_{\lambda }\right)\right]+\lambda \left[T_{1}\;S\left(\Phi \left(\rho \right)∥\Phi \left(\sigma_{\lambda }\right)\right)-T_{0}S\left(\rho ∥\sigma_{\lambda }\right)\right]\\ \; & \leq \left(1-\lambda \right)\left(T_{1}-T_{0}\right)S\left(\sigma ∥\sigma_{\lambda }\right)+\lambda \left(T_{1}-T_{0}\right)S\left(\rho ∥\sigma_{\lambda }\right)\leq 0\;. \end{array}$$

The first inequality uses the monotonicity of relative entropy [31], and the second inequality uses the assumption that $T_{1}\leq T_{0}$. Since our derivations holds for all ρ,σ,λ, the function G is concave everywhere. □

**Proof of Theorem 4.** Suppose that σ is a maximizer and it does not have full support, and let ρ be any state with full support. If Φ(·) has full support for all inputs, then S[Φ(ρ)∥Φ(σ)]<∞. Then, observe that the directional derivative diverges, $D_{\rho -\sigma }G\left(\sigma \right)=\infty$, since $T_{0}\;S\left(\rho ∥\sigma \right)=\infty$ while all other terms in Equation (14) are finite. The strict positivity (actually infinity) of the directional derivative contradicts the assumption that σ is a maximizer. Next, consider the case when $T_{0}>T_{1}$. Then,

$$T_{0}\;S\left(\rho ∥\sigma \right)-T_{1}\;S\left[\Phi \left(\rho \right)∥\Phi \left(\sigma \right)\right]\geq \left(T_{0}-T_{1}\right)S\left(\rho ∥\sigma \right)=\infty ,$$

where we used the monotonicity of relative entropy [31]. We again have $D_{\rho -\sigma }G\left(\sigma \right)=\infty$ from Equation (14), contradicting the assumption that σ is a maximizer. □
